# Thioredoxin-mimetic peptide attenuates epilepsy progression and neurocognitive deficits

**DOI:** 10.1016/j.redox.2026.104021

**Published:** 2026-01-10

**Authors:** Prince Kumar Singh, Shweta Maurya, Aseel Saadi, Sereen Sandouka, Taige Zhang, Orya Kadosh, Yara Sheeni, Valeria Martin, Daphne Atlas, Tawfeeq Shekh-Ahmad

**Affiliations:** a The Institute for Drug Research, The School of Pharmacy, Faculty of Medicine, The Hebrew University of Jerusalem, Jerusalem, - 91120, Israel; b The Alexander Silberman Institute of Life Science, The Hebrew University of Jerusalem, Jerusalem, Israel

**Keywords:** Epilepsy, Oxidative stress, Neuroinflammation, CB3 peptide, Disease-modifying therapy

## Abstract

Epilepsy is a chronic neurological disorder characterized by recurrent seizures, in which oxidative stress and neuroinflammation play central roles in driving disease progression and pharmacoresistance. Approximately 30–40 % of patients are resistant to current antiseizure medications, which suppress symptoms but do not prevent epilepsy development or modify its progression. There is an urgent need for therapies with true disease-modifying potential. TXM-CB3 (CB3), a thioredoxin-mimetic tripeptide, has been reported to modulate redox and inflammatory pathways. In this study, we evaluated the therapeutic potential of CB3 in preclinical models of temporal lobe epilepsy, focusing on its capacity to suppress seizures, preserve neuronal integrity, and mitigate epilepsy-associated behavioral impairments.

We first examined CB3 in an in vitro model of low-Mg^2+^-induced epileptiform activity, where pretreatment with CB3 (50, 100 μM) attenuated oxidative activity and reduced proinflammatory cytokine expression (IL-6, IL-1β, TNF-α), while enhancing IL-10 levels. In vivo, early CB3 intervention (20 mg/kg/day, i.p.) following kainic acid-induced status epilepticus significantly delayed seizure onset, reduced seizure frequency and cumulative burden, and preserved hippocampal neuronal integrity. Treated animals also showed improved locomotor activity, reduced anxiety-like behavior, and better performance in spatial working memory tasks. In established chronic epilepsy, CB3 treatment (20 mg/kg/day, i.p.) produced a sustained reduction in recurrent seizure activity and seizure burden, with additional effects on anxiety-like behavior, though memory and learning deficits remained unchanged.

Together, these findings highlight CB3's potential as a disease-modifying therapy. By reducing seizure recurrence, preserving neuronal integrity, and alleviating selected behavioral impairments, CB3 offers therapeutic benefits that extend beyond conventional ASMs and warrants further investigation for translation into clinical epilepsy treatment.

## 1 Introduction

Epilepsy is a prevalent, chronic neurological disorder characterized by recurrent, unprovoked seizures affecting individuals of all ages and backgrounds worldwide [1]. Despite the availability of antiseizure medications (ASMs), nearly 30–40 % of patients fail to respond to these FDA-approved treatments, leading to a growing population of drug-resistant epileptic patients [2,3]. Most of the available ASMs primarily target neuronal excitability but fail to address the underlying mechanisms that drive seizure recurrence and disease progression [4].

Among these mechanisms, oxidative stress and neuroinflammation are considered critical contributors to epileptogenesis, creating a self-perpetuating cycle that sustains neuronal hyperexcitability, promotes seizure-induced neuronal death, and exacerbates disease progression [[5], [6], [7], [8]]. Redox imbalance and overproduction of reactive oxygen species (ROS) promote mitochondrial dysfunction, neuronal excitability, and increase susceptibility to seizure activity [[9], [10], [11], [12]]. Both clinical and experimental studies have confirmed elevated indices of oxidative activity in epilepsy models [[13], [14], [15], [16]]. The redox disturbance promotes lipid peroxidation, protein oxidation, and DNA damage, creating a feed-forward loop in which oxidative stress amplifies hyperexcitability and exacerbates seizure-induced neuronal injury [11]. Although several antioxidant-based approaches have been tested in preclinical models, their translational success remains limited because of their inability to effectively disrupt the self-sustaining pathological cycle [17]. Our previous studies have highlighted the overexpression of NADPH oxidase 2 (NOX2) in preclinical models of epilepsy [12]. Importantly, we demonstrated that selective inhibition of NOX2 using both pharmacological and peptide-based approaches exerts potent anticonvulsive and disease-modifying effects in these models [[18], [19], [20]]. Such intervention was sufficient to confer neuroprotection within the hippocampus, attenuate recurrent seizure activity, and prevent the development of epilepsy-associated cognitive deficits [20]. These findings underscore the central role of oxidative stress in epileptogenesis and support redox modulation as a promising therapeutic strategy.

Inflammation represents another major driver of epileptogenesis [21]. Proinflammatory cytokines such as TNF-α, IL-6, and IL-1β alter synaptic plasticity, induce neuronal hyperexcitability, and impair blood‒brain barrier (BBB) integrity [21,22]. Previous studies confirmed that chronic neuroinflammatory responses are prevalent in drug-resistant epilepsy and contribute to progressive neuronal loss and cognitive impairment [[23], [24], [25]]. This proinflammatory milieu diminishes the seizure threshold by promoting glutamatergic excitotoxicity while concurrently impairing inhibitory neurotransmission, leading to elevated neuronal hyperexcitability [26,27]. Recent studies demonstrate that targeting inflammatory signaling markedly reduces neuronal pyroptosis, glial activation, and proinflammatory cytokine release in models of recurrent seizures [28,29]. Clinical and preclinical evidence further indicates that excessive inflammatory activity aggravates oxidative injury and cognitive comorbidities associated with epilepsy [28,29]. Collectively, these findings highlight the reciprocal interplay between neuroinflammation and oxidative stress in the epileptic brain, underscoring the need for therapeutic strategies capable of attenuating both pathological processes.

The thioredoxin reductase/thioredoxin (TrxR/Trx) system is a key regulator of redox homeostasis and neuroinflammatory signaling [[30], [31], [32], [33]]. It counteracts oxidative imbalance, limits proinflammatory cytokine release, and protects neurons from apoptosis. To harness these protective effects, thioredoxin-mimetic (TXM) peptides containing the redox motif Trx1-CxxC and Trx1-CxC were developed to mimic Trx activity [32,[34], [35], [36], [37]]. Within this family of tri- and tetra-TXM peptides, TXM-CB3 (Ac-CysProCys NH_2_) has been shown to suppress oxidative stress-induced inflammatory and apoptotic signaling, restore glutathione levels, upregulate anti-inflammatory cytokines, i.e., IL-10, and protect against cognitive decline in the models of traumatic brain injury and other pathologies [[37], [38], [39]].

Building upon the growing interest in peptide-based therapeutics, several peptide interventions have recently been explored in epilepsy, including TrkB-modulating peptides that attenuate epileptogenesis after status epilepticus [40], neuropeptide Y-based constructs that limit seizure spread [41], and venom-derived antiseizure peptides with favorable behavioral safety profiles [42]. Although promising, these approaches engage distinct molecular targets and have not demonstrated broad disease-modifying effects across key dimensions of temporal lobe epilepsy, such as sustained seizure reduction, preservation of neuronal integrity, and mitigation of cognitive or anxiety-related impairments. In this context, thioredoxin-mimetic peptides represent a mechanistically distinct strategy centered on redox and inflammatory regulation; however, CB3 has not previously been evaluated in epilepsy. Thus, whether a thioredoxin-mimetic peptide can influence epileptogenesis, suppress chronic spontaneous seizures, or improve epilepsy-associated behavioral deficits remains unknown. The present study addresses this gap by investigating, for the first time, the therapeutic and potential disease-modifying effects of CB3 in both early and chronic stages of experimental temporal lobe epilepsy.

In this study, we evaluate the therapeutic potential of TXM-CB3 (CB3) peptide in epilepsy models. We first examine whether CB3 modulates oxidative activtiy and proinflammatory cytokines expression in an in vitro model of epileptiform activity. We then assessed its antiepileptogenic efficacy by administering CB3 early after status epilepticus and evaluating its effects on recurrence of spontaneous seizure, neuronal survival, and behavioural outcomes. Finally, we investiagted whether CB3 treatment in established epilepsy could reduce seizure burden and modify behavioural impairments. Although CB3 is known to interact with redox and inflammatory pathways, detailed mechanistic interrogation was not the primary aim of this work. Instead, the study focuses on functional therapeutic outcomes to determine whether CB3 may serve as a potential disease-modifying approach, particularly in the context of pharmacoresistant epilepsy.

## 2 Materials and methods

### 2.1 Primary cortical cell culture

Primary cortical neuroglial cultures were prepared from Sprague–Dawley rat pups (P0–P1; Hebrew University breeding colony, Jerusalem, Israel) as described previously [18]. Briefly, neocortical tissue was dissected in chilled Hank's balanced salt solution (HBSS; Sartorius, Germany), digested with 0.25 % trypsin (Sigma-Aldrich, USA) at 37 °C for 6–7 min, and neutralized with HBSS solution containing 20 % FBS (Gibco, USA). Following mechanical trituration, single-cell suspensions were plated onto poly-l-lysine coated (1 mg/mL; Sigma-Aldrich, USA) 19 mm coverslips and maintained in Neurobasal®-A medium (Gibco, Thermo Fisher Scientific, USA) supplemented with B-27 (Gibco, Thermo Fisher Scientific, USA) and 2 mM l-glutamine. Cultures were maintained at 37 °C in 5 % CO_2_ and used for experiments at 13–14 days in vitro (DIV), when synaptic maturation is established.

#### 2.1.1 Induction of in vitro epileptiform activity

All live-cell imaging experiments were performed at room temperature using HEPES-buffered artificial cerebrospinal fluid (aCSF) to maintain physiological conditions. The aCSF solution contained 125 mM NaCl, 2.5 mM KCl, 2 mM MgCl_2_, 1.25 mM KH_2_PO_4_, 2 mM CaCl_2_, 30 mM glucose, and 25 mM HEPES; the pH was adjusted to 7.4 with NaOH. In vitro epileptiform activity was induced by switching to a magnesium-free aCSF solution (low Mg^2+^ conditions) in which MgCl_2_ was omitted while CaCl_2_ concentration was maintained at 2 mM, a physiological concentration widely used in low-Mg^2+^ seizure models to permit efficient NMDA receptor activation and support synchronized network hyperexcitability [43,44]. Maintaining 2 mM Ca^2+^ ensures efficient NMDA receptor activation under Mg^2+^-free conditions, as the removal of extracellular Mg^2+^ relieves the voltage-dependent block of NMDA channels, promoting Ca^2+^ influx and synchronized neuronal network hyperactivity characteristic of epileptiform discharges. The successful induction of in vitro epileptiform activity under low Mg^2+^ conditions was confirmed by Fura-2AM Ca^2+^ imaging (Abcam, ab120873), as described previously [18,19], which demonstrated synchronized calcium oscillations typical of epileptiform network activity.

#### 2.1.2 Assessment of oxidative activity in cultured neurons

Intracellular oxidative activity was assessed using live-cell fluorescence imaging with dihydroethidium (DHE; 5 μM, Abcam). Cultured neurons were pretreated for 2 h with vehicle or CB3 (50 μM or 100 μM; >98 % pure, Novetide, Israel), followed by real-time DHE imaging at an excitation wavelength of 530 nm under either aCSF conditions or low Mg^2+^ conditions. To minimize phototoxicity and photobleaching, light exposure was restricted to image acquisition periods. Each experiment was performed in five independent cultures, with duplicate coverslips per condition.

#### 2.1.3 Live imaging and analysis

Live-cell imaging was conducted with a 20 × fluorite objective of an epifluorescence inverted microscope (Nikon ECLIPSE Ti2, Nikon Corporation, Japan) equipped with a xenon arc lamp (Cairn Research, Kent, UK) and a cooled CCD camera (Retiga; QImaging). Phototoxicity and photobleaching were minimized by restricting light exposure to image acquisition. A single-wavelength excitation was used to reduce light-induced cell damage. Images were acquired at 5-s intervals, and fluorescence intensity was quantified at 10- and 15-min. Data were normalized to aCSF controls and expressed as a percentage relative fluorescence.

#### 2.1.4 Quantitative real-time polymerase chain reaction (qRT-PCR)

At DIV 14, cultured cells were exposed to low-Mg^2+^ conditions for 2 h, either alone or with 100 μM CB3, after which total RNA was extracted via Tri-Reagent (Sigma-Aldrich, St. Louis, MO, USA). Following extraction, the RNA samples were stored at −80 °C until further use. The RNA concentration and purity were determined via a NanoDrop One^C^ spectrophotometer (Thermo Fisher Scientific). Complementary DNA (cDNA) synthesis was carried out with 1 μg of RNA template, oligo-dT15 primers, and the GoScript™ Reverse Transcription System (Promega, Madison, WI, USA). Quantitative real-time PCR (qRT-PCR) was performed via the CFX Connect Real-Time PCR Detection System (Bio-Rad, USA) in a 15 μL reaction volume with 7.5 μL of SYBR Green mix (PerfeCTa SYBR Green FastMix, Quantabio), 1.5 μL of DEPC-treated water (Sigma, USA), 3 μL of primer mixture (500 nM each), and 3 μL of cDNA template. The thermal cycling conditions included initial denaturation at 95 °C for 10 min; 40 cycles of denaturation at 95 °C for 5 s and 60 °C for 15 s; and a final step of 5 s at 65 °C and 30 s at 95 °C. Each qRT-PCR reaction was performed in duplicate. The primer sequences for the target and control genes were adapted from previous reports [19] as follows: IL-6, forward 5′-GACTTCCAGCCAGTTGCCTT-3′ and reverse 5′-CTGGTCTGTTGTGGGTGGTAT-3′; IL-1β, forward 5′-CCCTGCAGCTGGAGAGTGTGG-3′ and reverse 5′-AGCACCTCTCAAGCAGAGCACA-3′; IL-10, forward 5′-AATAAGCTCCAAGACAAAGGT-3′ and reverse 5′-CTGCATAGAAGCCTACGTGA-3′; and TNFα, forward 5′-ATGGGCTCCCTCTCATCAGT-3′ and reverse 5′-GCTTGGTGGTTTGCTACGA-3′. Gene expression levels were quantified relative to the control, GAPDH, via the method described by Pfaffl et al. (2001) [45]. The results are expressed as relative mRNA levels normalized to the aCSF condition.

### 2.2 Animal model and experiments

All animal procedures were conducted in accordance with the Association for Assessment and Accreditation of Laboratory Animal Care (AAALAC) International guidelines and were approved by the Institutional Animal Care and Use Committee (IACUC) of Hebrew University of Jerusalem (Approval No. MD-20-16254-5). Male Sprague-Dawley (SD) rats weighing 150–199 g from the Hebrew University strain (Harlan, Jerusalem, Israel) were used for this study. All animals were housed under regulated conditions (23 °C, 50–60 % humidity, 12-h light/dark cycle) with ad libitum access to food and water. Prior to experimentation, all animals were acclimatized for one week to ensure adaptation to laboratory conditions.

#### 2.2.1 Experimental grouping

In the cohort I study (assessment of the antiepileptogenic effect), 16 naïve male SD rats (175–195 g) were used. All 16 naïve male rats underwent ECoG implantation surgery, and one-week post-surgery, status epilepticus (SE) was induced via kainic acid (KA). One animal in the CB3-treated group did not survive the KA injection. All surviving SE animals were then randomly assigned to the vehicle or control (n = 8) group or CB3 treatment (n = 7) group and subjected to two weeks of therapy with either vehicle (saline) or CB3 beginning at 1 h after SE termination. All animals were continuously monitored 24/7 for vECoG activity for the next 10–12 weeks. At the end of the cohort I study, the animals were perfused for histological assessment of the brain.

In the cohort II study (chronic epilepsy model), 18 naïve male SD rats (150–175 g) were included. All animals were induced with status epilepticus and allowed to develop the chronic phase of epilepsy for the next 10–11 weeks. At 10–11 weeks post SE induction, the animals were implanted with an ECoG transmitter and continuously monitored 24/7 for recurrent seizure activity. From the total of 16 chronic phase animals, 12 animals confirmed recurrent seizure frequency and were included in the preclinical trial of cohort II. All confirmed animals were randomly assigned to either the vehicle-treated (n = 6) or CB3-treated (n = 6) group and received two weeks of their respective therapy, followed by four weeks of post-therapy seizure monitoring.

#### 2.2.2 Animal surgery: implantation of an ECoG transmitter

All animals in cohorts I and II underwent electrocorticography transmitter (ECoG) implantation surgery under general anesthesia. Anesthesia was induced with 3 % isoflurane and maintained at 1.8–2.3 % isoflurane (Terrell™, USP, Piramal Critical Care). The animals were securely positioned in a stereotaxic frame (Kopf, CA, USA). Presurgical analgesics, including buprenorphine (0.2 mg/kg, s.c.; Rich Pharma) and meloxicam (1 mg/kg, s.c.; Chanelle Pharma), were administered. To ensure perioperative analgesia, buprenorphine (0.2 mg/kg, s.c.; Rich Pharma) and meloxicam (1 mg/kg, s.c.; Chanelle Pharma) were administered prior to surgery. The animals were subcutaneously implanted with an ECoG transmitter (A3028E, single channel, 512 Hz, Open-Source Instruments, Inc.) equipped with two subdural intracranial electrodes. The recording electrode was placed above the right hippocampus at AP: 3 mm, ML: +2.5 mm, whereas the reference electrode was positioned in the contralateral hemisphere at AP: 6/-7 mm, ML: +3.5 mm. The electrodes were secured via skull screws and tissue glue to ensure stable signal acquisition. Following implantation, the cranial incision was sealed with dental cement, and each animal received 3–5 ml of warmed Ringer's solution, along with amoxicillin (100 mg/kg; Betamox LA), to prevent postsurgical infection. The animals were returned to their home cages for a one-week recovery period.

#### 2.2.3 Induction of status epilepticus

All male SD rats (175–200 g) were subjected to status epilepticus (SE) induction using the intraperitoneal administration of kainic acid (KA; 5 mg/ml in 0.9 % saline; *i.p.;* Hello-bio), as previously described [46,47]. An initial dose of 10 mg/kg KA was administered, and animals were closely monitored for seizure progression according to the modified Racine's scale [48]. If stage IV-V seizures were not observed following the first injection, subsequent hourly doses of 2.5 mg/kg KA were administered until the animals exhibits class IV-V seizures, characterized by rearing, forelimb clonus, and falling. All animals developed stage V seizures with a cumulative KA dose ranging from 10 to 15 mg/kg. Two hours after confirming SE onset (persistent class V seizures), diazepam (10 mg/kg; Assival®, Teva Pharmaceutical Industries Ltd., Israel) was administered intraperitoneally to terminate seizure activity and reduce mortality. Rats that continued to exhibit continuous motor seizures during the 2-h post-SE period or after the final KA dose were selected for further experimentation.

#### 2.2.4 Drug administration

In the cohort I study (assessment of the antiepileptogenic effect), animals that underwent SE induction were randomly assigned to either the vehicle (n = 8) or CB3 treatment (n = 7) group 1 h after diazepam administration (SE termination). CB3 was freshly dissolved in sterile normal saline (0.9 % NaCl, pH 7.2–7.4; 20 mg/mL) and administered intraperitoneally at a dose of 20 mg/kg/day, corresponding to an injection volume of 1 mL/kg body weight, for two weeks. Control animals receive an equivalent volume of vehicle (saline) following the same schedule.

In the cohort II study (chronic epilepsy model), animals at 10–11 weeks post-SE induction that exhibited recurrent spontaneous seizures, confirmed by continuous 24/7 video-ECoG (vECoG) monitoring during four consecutive weeks of baseline recording, were considered to have reached the chronic epilepsy stage. These animals were then randomized into either the vehicle (n = 6) or CB3 treatment (n = 6) group and received the same two-week dosing regimen as described in cohort I.

#### 2.2.5 ECoG data acquisition and analysis

All animals in the present study were implanted with subdural ECoG transmitters and housed individually under continuous 24/7 wireless vECoG surveillance. Wireless ECoG telemetry was controlled and recorded via Neuroarchiver software (Open-Source Instruments Inc.), ensuring high-fidelity data acquisition and real-time analysis. The recorded ECoG data were segmented into 4-s epochs, and six key metrics were analyzed: power, intermittency, coastline, coherence, asymmetry, and power within the 12–30 Hz range. Specifically, power was calculated as the mean squared amplitude of the signal; intermittency as the ratio of burst duration to interburst interval; coastline as the cumulative absolute voltage change per epoch; coherence as the normalized cross-spectral density between channels; asymmetry as the normalized amplitude difference between hemispheric signals; and power in the 12–30 Hz band was determined using fast Fourier transform (FFT)-based spectral analysis. Each metric was normalized to a 0–1 interval and compared with a user-generated seizure library containing verified seizures from at least three different animals. Automated seizure detection was performed using an algorithm that classified epochs as seizure-like events if their Euclidean distance was less than 0.2 from a validated seizure epoch based on predefined event detection criteria (Open Source Instruments, Event Detection Guidelines). Automated screening served as an initial step; final seizure identification was based on electrographic characteristics; including abnormally high-amplitude activity (>2 × baseline) with rhythmic discharges and evolution in frequency and/or amplitude lasting ≥10 s, followed by post-ictal suppression. All detection events were manually reviewed and confirmed by a treatment-blind researcher with a subset further validated through synchronized video monitoring via digitally time-locked CCTV cameras (Microseven) positioned outside the cages. Video recordings were assessed by a different blind researcher to ensure the correlation between behavioral and electrographic seizure activity for enhanced accuracy in seizure classification. This combined automated-manual ensured methodological rigor, accuracy, and reproducibility in seizure detection and classification.

### 2.3 Tissue collection and sample preparation

The animals were sacrificed under deep anesthesia via the intraperitoneal administration of ketamidor (100 mg/kg; Richter Pharma AG) and sedaxylan (10 mg/kg; Eurovet Animal Health B.V.). Once fully anesthetized, the animals underwent transcardial perfusion using a perfusion system (MRC Laboratory Instruments Ltd., UK) at a constant flow rate of 9 mL/min. Perfusion was performed first with 1 × PBS containing heparin (200–250 mL) to clear the vasculature, followed by 4 % formaldehyde in 1 × PBS (250–300 mL total volume; 37 %, Sisco Research Laboratories, SRL) to fix the tissue. At this flow rate, the total perfusion duration was approximately 55–65 min. Following perfusion, brain samples were carefully extracted and fixed overnight in 4 % PFA at 4 °C, followed by cryoprotection in a sucrose gradient (10 %-20 %–30 %) for 3–4 days until the tissue sank. After cryoprotection, the brains were embedded in OCT compound (Scigen) and stored at −80 °C until further processing. For histological analysis, brain sections were prepared via a cryostat (Leica CM1950) at −20 °C. Sections were cut to a thickness of 25 μm and mounted onto poly l-lysine-coated slides (Thermo Scientific, USA) for subsequent staining and imaging.

### 2.4 Immunohistochemistry and microscopy

The brain sections were outlined with a water-repellent pen (Dako pen; Agilent) and washed with 1x PBS (Sigma‒Aldrich, USA) for 1 min. The sections were permeabilized for 30 min in 0.2 % Triton X-100 (Sigma‒Aldrich, USA) in 1x PBS, followed by blocking for 2 h in 7 % goat serum (Vector Laboratories) containing 0.1 % Triton X-100 in 1 % BSA (MP Biomedicals) in 1x PBS. After blocking, the sections were washed three times with 1x PBS (10 min each wash). The washed sections were incubated overnight at 4 °C with a primary antibody mixture prepared in 0.1 % Triton X-100 in 1 % BSA in 1x PBS. The primary antibody mixture contained an anti-NeuN (rabbit, 1:500; ab177487) antibody for neurons and an anti-8OH'dG antibody (mouse, 1:500; ab62623) for DNA damage. Following overnight primary antibody incubation, the sections were washed (as above) in 1x PBS and then incubated for 2 h with the following secondary antibody mixture: Alexa Fluor®-488 goat anti-rabbit (1:500, ab150081) and Alexa Fluor®-568 goat anti-mouse (1:500, ab175701) in the dark. Following secondary antibody incubation, the sections were washed (as above) in 1x PBS, allowed to dry in the dark and mounted with DAPI mounting medium (Abcam). Immunohistochemical images were acquired via the Nikon 10× objective of a confocal A1R confocal microscope. Image analysis was performed via the NIS Elements Advanced Research Software. The mean intensity was calculated and normalized to the average of vehicle-treated animals. The results are expressed as the mean fluorescence intensity (A.U.), normalized to that of the vehicle.

### 2.5 Nissl staining and imaging

Neuronal degeneration in the hippocampus was evaluated via Nissl staining to visualize neuronal degeneration. Coronal brain sections with a thickness of 25 μm were first dried and placed in 1:1 alcohol/chloroform overnight, followed by rehydration with a graded alcohol series of 100 %, 95 %, and distilled water, each for 5 min. The rehydrated sections were stained with 0.1 % cresyl violet (Thermo Scientific, USA) solution at room temperature for 5–10 min and rinsed with distilled water to remove excess stains. Following washing, the sections were differentiated in 95 % alcohol for ∼30 min, or until the background appeared pale while neuronal somata remained clearly defined, as confirmed by brief microscopic inspection. The sections were then dehydrated with absolute alcohol (twice, 5 min each), cleared in xylene (twice, 5 min each), and mounted with Eukitt® Quick-Hardening Mounting Medium (Sigma-Aldrich, USA). Bright-field images were captured with a 20× objective of the SideView VS200 digital slide scanner (VS200 Multiple Tray Loader, Olympus) under bright-field illumination, controlled via Olympus VS200 software. For regional analysis, the CA1 and CA3 subfields of the dorsal hippocampus were identified based on their anatomical landmarks and coordinates according to the rat brain stereotaxic atlas [49]. Image analysis was performed manually to quantify the Nissl-stained neurons in the CA1 and CA3 subfields of the hippocampus. The results are expressed as the percentage of neuronal density per mm^2^ normalized to vehicle density in both hippocampal subfields.

### 2.6 Behavioral assessment

All ECoG-implanted animals from cohorts I and II underwent behavioral assessment to evaluate the effect of CB3 therapy on epilepsy-associated cognitive decline. Locomotor activity and anxiety-like behavior were assessed via the open field (OF) test and elevated plus-maze (EPM) test. Novel object recognition (NOR) and T-maze alteration tests were performed to determine the effects of CB3 therapy on recognition memory and spatial learning.

#### 2.6.1 Open field (OF)

The open field (OF) arena consisted of a plexiglass box measuring 100 × 100 × 40 cm. The central zone (50 × 50 cm) represented 25 % of the total floor area, a commonly used proportion to evaluate anxiety-like behavior. Each animal was placed in the arena and allowed to explore freely for 10 min while being recorded by an overhead CCTV camera. Video recordings were analyzed via EthoVision XT software, which measures the total distance traveled, percentage time spent in the center zone (50 × 50 cm), speed (cm/s), total movement duration, and freezing duration (in seconds). The OF arena was thoroughly cleaned with 70 % ethanol between trials to eliminate residual olfactory cues.

#### 2.6.2 Elevated plus maze (EPM)

The EPM consisted of two open arms (50 × 12 cm) and two enclosed arms (50 × 12 cm) extending from a central platform (12 × 12 cm) elevated 50 cm above the floor. The day before the experiment, each animal was placed at the center of the maze and allowed to explore for approximately 10 min. On the assessment day, each animal was placed at the intersection of the open and closed arms, and a 10-min test session was performed. During the test, the animals were allowed to explore either the open or closed arms freely. All movements were recorded with an overhead CCTV camera, and the CCTV footage was analyzed via a semiautomatic tracking script in DeepLabCut™ (https://github.com/DeepLabCut) [50] to determine the primary behavioral parameters, such as the total number of entries and percentage of time spent in the open arms. The maze was cleaned with 70 % ethanol between trials.

#### 2.6.3 Novel object recognition (NOR)

The NOR test was conducted in the same open-field arena (100 × 100 × 40 cm). During the familiarization phase, each animal was placed in an arena containing two identical objects (500 mL Borosil® glass bottles; diameter ∼86 mm and height ∼180 mm) wrapped with opaque white laboratory tape to minimize reflections and visual bias, and allowed to explore for 10 min. After a 90-min delay, one of the familiar objects was replaced with a novel object (a closed, non-porous polypropylene pipette tip box of 200 μL capacity; approximately 120 × 83 × 65 mm), also wrapped with opaque white tape to ensure consistent surface appearance and the animal was reintroduced to the arena for a 10-min recognition session. Exploration behavior was recorded via an overhead CCTV camera, and video analysis was performed via EthoVision XT software to determine the discrimination index (DI) and exploration frequency. To prevent olfactory bias, the arenas and objects were cleaned with 70 % ethanol between trials. The object presentation was counterbalanced to control for side preference.

#### 2.6.4 Spontaneous alternation T-maze

The T-maze consisted of a symmetrical T-shaped structure with a central stem and two perpendicular goal arms (50 × 12 cm). Each trial began with the animals being placed in the start box and allowed to navigate through the stem and choose either the left or right arm. A choice was recorded when the animal entered the goal arm with all four limbs. Upon arm entry, a guillotine door was closed for 30 s, after which the animal was returned to the start box for the next trial. The intertrial interval was 1 min to maintain consistency between trials. The percentage of correct alterations was calculated via the following formula: (total number of correct alternations/6) × 100. A higher alternation percentage indicates better spatial working memory. All behavioral assessment experiments were conducted by an experimenter blinded to the treatment groups.

### 2.7 Statistical analysis

Statistical analyses were performed via GraphPad Prism v9.3.1 (GraphPad Software, USA). Data acquisition and analysis were performed in a blinded manner. The quantitative results are expressed as the means ± standard errors of the means (SEMs), with n denoting the number of samples. Data normality was assessed using the Shapiro-Wilk and Kolmogorov-Smirnov tests. Accordingly, parametric tests (unpaired Student's t-test for pairwise comparisons and one-way or two-way ANOVA with Tukey's post hoc test for multiple group comparisons) or non-parametric tests (Mann-Whitney) were applied as appropriate. A mixed-effects model was used for the comparison of seizure frequency. Sample sizes were selected on the basis of prior experience and estimates of experimental variability to ensure reliable statistical analysis. Statistical significance was set at p < 0.05, with additional significance thresholds set at p < 0.01. The number of animals used in each experiment is specified in the respective figure legends.

## 3 Results

### 3.1 CB3 attenuates oxidative activity and proinflammatory cytokine response during epileptiform activity

**Fig. 1:**
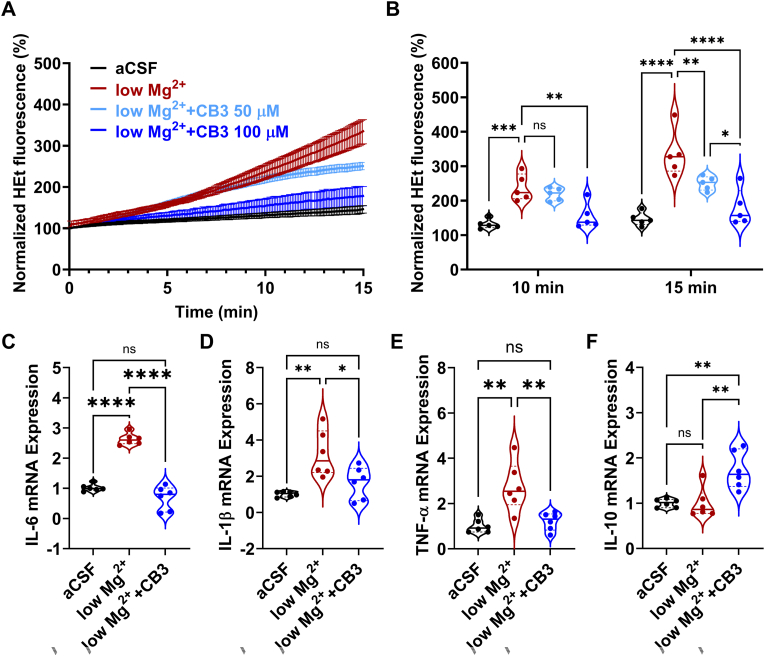
**CB3 modulates oxidative activity and cytokine expression in low Mg^2+^-induced epileptiform activity.** (A) Time-dependent normalized HEt fluorescence intensity in aCSF (black) and low Mg^2+^ condition without treatment (red), and with CB3 pretreatment at 50 μM (light blue) and 100 μM (blue). (B) Quantification of normalized HEt fluorescence at 10 min and 15 min n = 5 independent experiments with duplicate coverslips. (C–F) Relative mRNA expression level of IL-6 (C), IL-1β (D), TNFα (E), and IL-10 (F) measured after 2 h of exposure to aCSF or low Mg^2+^ condition, with and without CB3 pretreatment. n = 6 independent experiments with duplicate coverslips. Data are presented as mean ± s.e.m. Statistical significance was determined by one-way or two-way ANOVA followed by post hoc Tukey's test. ∗p < 0.05, ∗∗p < 0.01, ∗∗∗p < 0.001, ∗∗∗∗p < 0.0001, ns: not significant.

We first assessed the effect of CB3 in an in vitro epileptiform activity model. We utilized the widely used low-magnesium (Mg^2+^) paradigm, in which omitting Mg^2+^ from the culturing medium, triggers N-methyl-d-aspartate (NMDA) receptor-dependent network hyperexcitability that recapitulates key cellular features of seizure-like activity [44]. Consistent with previous reports [18,43,51], exposure of primary cortical cultures to Mg^2+^-free medium induced a robust increase in HEt fluorescence reaching ∼336 % of baseline within 15 min, reflecting enhanced oxidative activity (Fig. 1A and B). Pretreatment with CB3 (50 μM or 100 μM, 2 h) significantly attenuated this fluorescence response in a concentration-dependent manner, reducing signal to ∼250 % and ∼180 % of baseline, respectively (Fig. 1B).

We next examined the inflammatory changes after 2 h of low-Mg^2+^ exposure. Under same conditions, low-Mg^2+^ exposure significantly induces the expression of proinflammatory cytokines, such as IL-6, IL-1β, and TNF-α, at the mRNA level (Fig. 1C–E), while leaving anti-inflammatory cytokine IL-10 unchanged (Fig. 1F). However, CB3 pretreatment (100 μM, 2 h) significantly inhibits proinflammatory cytokines IL-6, IL-1β, and TNF-α expression (Fig. 1C–E, respectively), and concomitantly enhanced anti-inflammatory cytokine IL-10 expression (Fig. 1F). These findings align with earlier studies showing that CB3 exerts protective effects by modulating oxidative and inflammatory responses in cardiovascular disease [52,53], diabetes [32], radiation [54], traumatic brain injuries [39,55], and several other pathological conditions [33,38,56,57].

### 3.2 C*B3 inhibits epilepsy development and delays recurrent seizure activity following SE*

**Fig. 2:**
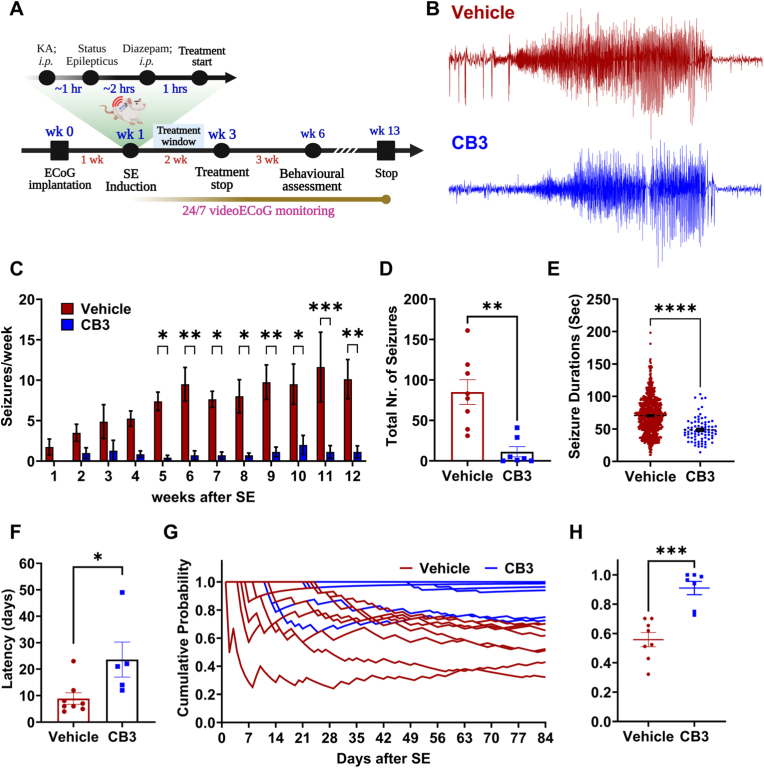
**CB3 suppresses epilepsy development and overall seizure burden following SE.** (A) Experimental timeline for cohort I animals illustrating ECoG implantation, SE induction, treatment window, behavioral assessments, and continuous 24/7 video-ECoG monitoring across the 12-week study period. (B) Representative EEG traces from Vehicle-treated (red) and CB3-treated (blue) animals showing typical spontaneous seizures. (C) Bar graph showing seizure frequency per week in control (red) and CB3-treated (blue) groups over a 12-week duration after SE. (D) Total number of seizures experienced by each animal over 12 weeks duration. (E) Seizure durations (in seconds) for all scored seizures in Vehicle versus CB3-treated animals. (F) Latency to the first spontaneous seizure (in days) following SE induction for each animal. (G) Cumulative probability of seizure-free days in individual animals from vehicle (red) and CB3 (blue) treatment over 84 days. Each line represents an individual animal's seizure-free trajectory. (H) The cumulative probability of seizure-free days in vehicle and CB3-treated animals in G. In Vehicle animals (n = 8); CB3-treated animals (n = 7). Data are presented as mean ± s.e.m. Statistical significance was determined by generalized log-linear mixed model, incorporating the random effect of the animal (autoregressive covariance) and fixed effects of treatment group, time, and the interaction between treatment group and time (panel C): *F*(11, 143) = 2.157, *p* = 0.0199); unpaired *t*-test (panels D, F); by Mann-Whitney test (H). ∗p < 0.05, ∗∗p < 0.01, ∗∗∗p < 0.001, ∗∗∗∗p < 0.0001, ns: not significant.

Next, we assessed the long-term effect of CB3 treatment on epilepsy development and progression. We administered kainic acid to induce status epilepticus in all animals from cohort I (Fig. 2A) and randomly allocated them into vehicle (n = 8) and CB3 treatment (n = 7) groups. As expected, we did not observe any differences between groups in any SE induction parameter (Supp Fig. 1). SE induction was verified with vECoG monitoring (Supp Fig. 1A), and both SE duration (Supp Fig. 1B; p = 0.3829) and latency to SE onset (Supp Fig. 1C; p = 0.7926) were comparable between Vehicle- and CB3-assigned animals, confirming unbiased group allocation and equivalent initial insult severity (Supp Fig. 1A–C). Following SE, all animals underwent continuous video-ECoG monitoring, and CB3 or vehicle treatment was administered for two weeks, beginning 1 h post SE (Fig. 2A). During the 12-week epilepsy vECoG monitoring duration (Fig. 2A), vehicle-treated animals exhibited a progressive increase in spontaneous seizure frequency, reaching maximal seizure burden during weeks 10–12 post-SE (Fig. 2C). In contrast, CB3-treated animals showed a robust and sustained reduction in seizure frequency throughout the monitoring period (Fig. 2C), suggesting that early intervention with CB3 for two weeks is sufficient to maintain this long-lasting antiepileptogenic effect. To evaluate seizure progression within each group, we performed week-to-week comparisons. Vehicle-treated animals displayed a significant escalation in seizure frequency beginning at week 3 and continuing through week 12 (p < 0.05 to p < 0.0001), whereas CB3-treated animals showed no significant temporal variation (p > 0.99), indicating stabilization of seizure activity (Fig. 2C, mixed-effects analysis with Dunnett's and Tukey's post hoc tests). Furthermore, the total seizure burden, quantified as the cumulative number of seizures experienced by each animal, was significantly lower in CB3-treated animals than in the vehicle group (Fig. 2D, p < 0.01). Compared with the vehicle group, CB3 treatment experience significantly lower seizure duration (Fig. 2E, p < 0.0001) with significantly prolonged latency to the first spontaneous seizure (Fig. 2F; p < 0.05). Cumulative probability plots further demonstrated a rapid decline in seizure-free status in vehicle-treated animals, whereas CB3-treated animals maintained a significantly higher probability of remaining seizure-free across the 84-day period (Fig. 2G–H; p < 0.001). Together, these findings indicate that early CB3 therapy delays epilepsy onset, reduces chronic seizure burden, and prevents the progressive escalation of spontaneous seizures following SE, supporting its strong antiepileptogenic potential.

### 3.3 CB3 prevents cognitive decline during epilepsy development

**Fig. 3:**
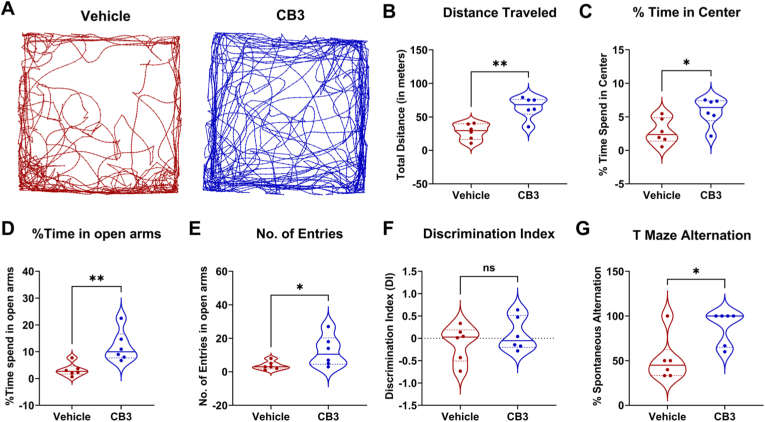
**CB3 prevents cognitive declines associated with epilepsy development.** (A) Representative track path of vehicle (red) and CB3 (blue) treated animals in open field arena at fifth week after SE (third week after therapy withdrawal). Open Field test: Total distance traveled (B), Percentage time spent in center (C) by vehicle and CB3 treated animals. Elevated plus maze test: Percentage time spent in open arms (D), No. of entries in open arms (E) by vehicle and CB3 treated animals. Novel object recognition test showing discriminative index (F) in vehicle and CB3 treated animals. T-maze spontaneous alternation test showing percentage of correct alteration (G) by vehicle and CB3 treated animals. n = 6 animals per group. Data are presented as mean ± s.e.m. Statistical significance was determined by unpaired *t*-test. ∗p < 0.05, ∗∗p < 0.01, ns: not significant.

To evaluate the effects of CB3 treatment on cognitive function and anxiety-like behavior, we assessed behavioral performance at the fifth week after SE (third week after CB3 withdrawal) in animals from cohort I. The open field (OF) test, conducted in an arena divided into a central zone (50 × 50 cm; 25 % of total arena area) to distinguish exploratory and anxiety-related behavior. CB3 treatment improved locomotor activity, as evident by a significantly greater total distance traveled (Fig. 3B, p < 0.01) and higher average movement speed (10.74 ± 1.10 cm/s vs 4.73 ± 0.76 cm/s, CB3 vs vehicle; p < 0.01) compared to the vehicle-treated group. Moreover, CB3-treated animals entered the central area more frequently (16.67 ± 1.26 vs 5.83 ± 1.37, CB3 vs vehicle; p < 0.01) and spent a higher percentage of time in the center zone (Fig. 3C, p < 0.05), indicating enhanced locomotor activity and reduced anxiety-like behavior. These results were further supported by their arena exploration track paths (Fig. 3A), suggesting that CB3-treated animals were less anxious and explored the arena more freely compared to vehicle-group. We further confirmed this reduced anxiety-like behavior in the elevated plus-maze (EPM) test. Our findings revealed that CB3-treated animals spent significantly more time in the open arms (Fig. 3D, p < 0.01) with significantly higher entries into the open arms (Fig. 3E, p < 0.05) than vehicle-treated animals. We further examined the impact of CB3 on cognitive performance by assessing recognition memory via the novel object recognition (NOR) test. No significant differences were observed in the discrimination index (DI, Fig. 3F) between the two groups. However, in the T-maze spontaneous alternation test, CB3-treated animals showed significantly improved performance (Fig. 3G, p < 0.05), suggesting a protective effect on spatial working memory. These results suggest that CB3 preserves motor and cognitive functions rather than general exploratory behavior. The lack of difference in the NOR test may reflect the task's limited sensitivity to mild recognition memory deficits and possibly that CB3's effects are more prominent in hippocampal-dependent spatial memory than in perirhinal cortex-mediated object recognition.

### 3.4 CB3 protects from seizure-induced oxidative DNA damage in hippocampal neurons

Seizure activity is known to induce oxidative stress and DNA damage in hippocampal neurons, contributing to neuronal vulnerability and epileptogenesis [9,58,59]. Among oxidative lesions, 8-hydroxy-2′-deoxyguanosine (8-OHdG) is widely used as a marker of oxidative DNA modification in both preclinical and clinical studies [[60], [61], [62]].

Given CB3's sustained reduction in recurrent seizure activity (Fig. 2), we examined whether this was accompanied by changes in oxidative DNA damage in hippocampal neurons. We assessed oxidative DNA damage via 8-OHdG (a marker for oxidative stress-mediated DNA damage) immunostaining co-localized with NeuN-positive neuronal population in the CA1 and CA3 subfields of vehicle- and CB3-treated animals (Supp Fig. 2A and B). CB3-treated animals exhibited a significant reduction in 8-OHdG fluorescence intensity in CA1 neurons compared with vehicle-treated animals (Supp Fig. 2A and C; p < 0.05), suggesting decreased oxidative DNA damage. However, no significant differences were observed in the CA3 subfield (Supp Fig. 2B and D). These findings provide supportive evidence that CB3 may attenuate seizure-associated oxidative stress in vulnerable hippocampal regions.

### 3.5 CB3 protects neuronal integrity and reduces seizure-induced neuronal loss in the hippocampus

**Fig. 4:**
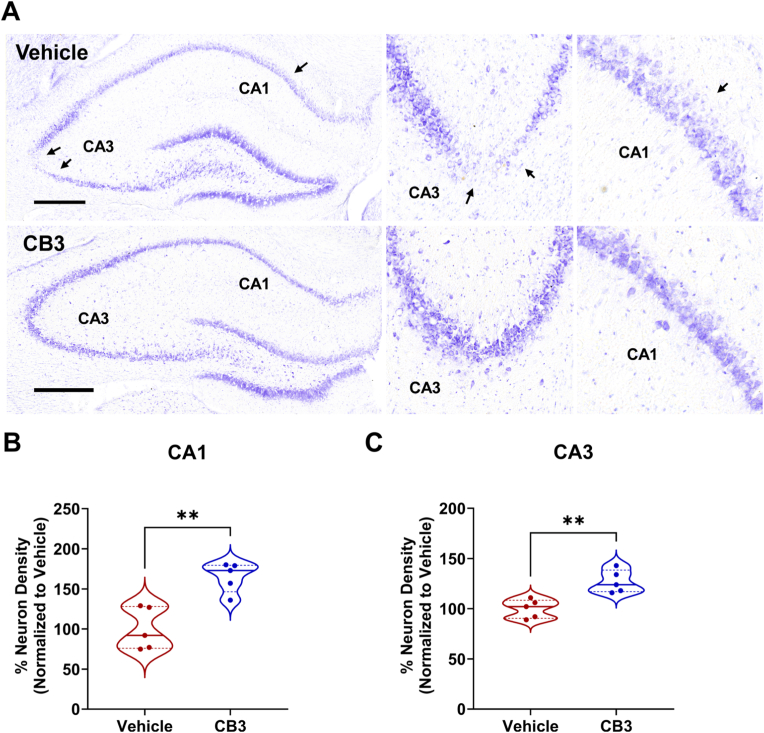
**CB3 exert a sustained protective effect on hippocampal neurons following recurrent seizure activity.** (A) Representative Nissl-stained images showing hippocampal subfield CA1 and CA3 of vehicle and CB3 treated animals after SE, followed by 12 weeks of vECoG monitoring. Higher magnification images of CA1 (middle) and CA3 (right) panels. Arrows indicate neuronal degeneration. (B, C) Quantification of neuronal density in the CA1 (B) and CA3 (C) subfields of the hippocampus. n = 5 animals per group. Scale bar is 500 μM. Data are presented as mean ± s.e.m. Statistical significance was determined by unpaired *t*-test. ∗∗p < 0.01.

We next examined the impact of early CB3 intervention after SE on recurrent seizure activity-induced neuronal death and structural integrity during the late phase of epileptogenesis. Compared with vehicle-treated animals, CB3-treated animals showed reduced neuronal loss in both the CA1 (Fig. 4A and B) and CA3 (Fig. 4A–C) subfields of the hippocampus. This protective effect was reflected in higher neuronal density in the CB3-treated animals compared to vehicle group (Fig. 4B–C, p > 0.01). However, the neuronal loss associated with the development of epilepsy is not limited to a reduction in neuronal density. We also observed distortion of neuronal integrity in the CA3 subfield (Fig. 4A) of vehicle-treated animals, whereas early intervention with CB3 prevented the associated distortion of neuronal integrity.

Taken together, these findings suggest that CB3 not only suppresses seizures but also limits neuronal loss and structural disruption during epilepsy development. In addition, CB3-treated animals exhibited improved cognitive outcomes, supporting its potential as a disease-modifying therapy that addresses both seizure burden and associated cognitive impairments.

### 3.6 CB3 therapy modifies chronic epilepsy

**Fig. 5:**
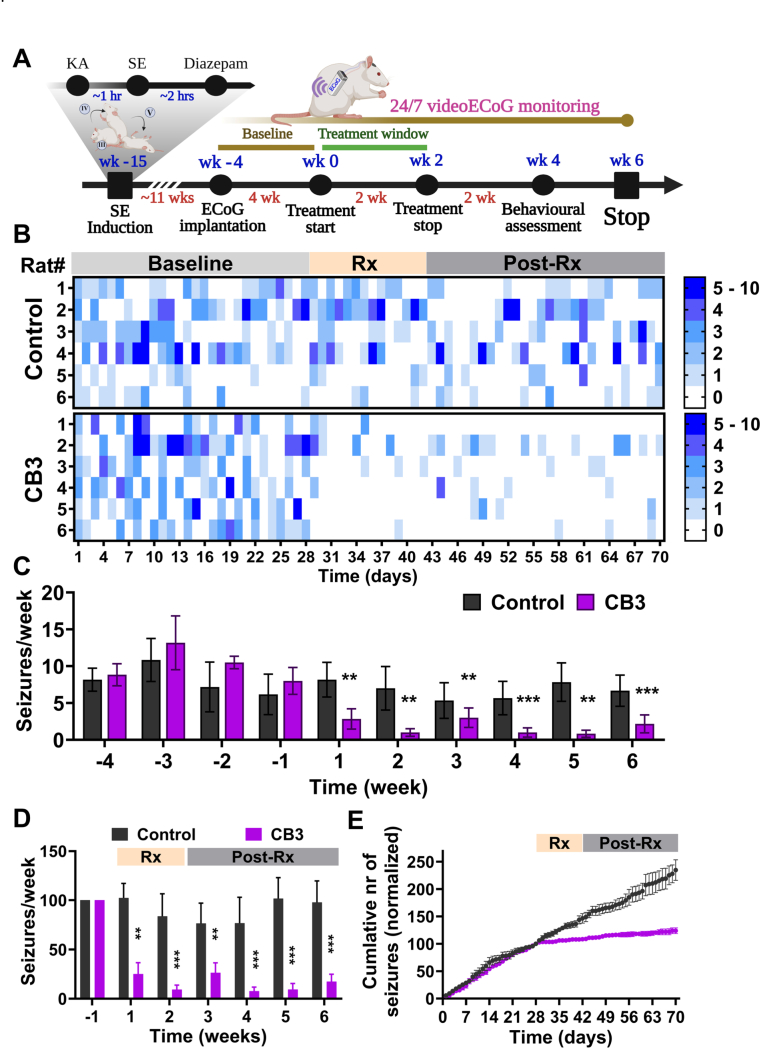
**CB3 treatment suppresses recurrent seizure activity and seizure progression in epileptic animals.** (A) Experimental timeline for Cohort II animals illustrating the induction of SE with KA and diazepam intervention, ECoG implantation, followed by a baseline recording and treatment window, behavioral assessments, and continuous 24/7 video-ECoG monitoring throughout the study. (B) Raster plot showing seizure frequency in control (top) and CB3-treated (bottom) animals of chronic phase across the duration of pre-clinical trial. The baseline (before treatment, 4 weeks), treatment (Rx, 2 weeks), and post-treatment (post-Rx, 4 weeks) phases are indicated. (C) Weekly seizure frequency in control (black) and CB3-treated (purple) animals of chronic phase. (D) Total weekly seizure burden in baseline and during treatment and after treatment. (E) Cumulative number of seizures experienced by animals in chronic phase epilepsy. n = 6 animals per group. Data are presented as mean ± s.e.m. Statistical significance was determined by generalized log-linear mixed model, incorporating the random effect of the animal (autoregressive covariance) and fixed effects of treatment group and time (C–D). ∗∗p < 0.01, ∗∗∗p < 0.001, ns: not significant.

Building on the therapeutic effects observed with early intervention, we next evaluated CB3 treatment in animals with fully established epilepsy. In the second cohort, SE was induced with KA, and animals were allowed to progress to the chronic phase of epilepsy, characterized by recurrent spontaneous seizure activity confirmed by vECoG monitoring (Fig. 5A and B). Epileptic animals were then randomized to receive either vehicle or CB3 treatment. CB3 treatment significantly suppresses the spontaneous recurrent seizure activity (Fig. 5A–D), and this seizure-suppressive effect persisted throughout the observation period. By week 6, seizure frequency in CB3-treated animals was reduced by more than 60 % compared to control group (Fig. 5B–D, p < 0.001). In addition, recurrent seizure activity declined from week 2 onward in the CB3 group (Fig. 5C, p < 0.01), consistent with a lower overall seizure burden (Fig. 5C). The cumulative seizure burden further confirmed that CB3-treated animals experienced fewer seizures than control animals across the study period (Fig. 5D). Differences in seizure progression between groups emerged during the treatment phase and were sustained throughout the posttreatment period. Our results indicate that CB3 treatment reduces seizure frequency and cumulative burden in the chronic phase of epilepsy, with effects persisting after treatment withdrawal. These data support CB3's potential to modify the course of established epilepsy.

### 3.7 CB3 modulates anxiety-related behavior in chronic epilepsy

**Fig. 6:**
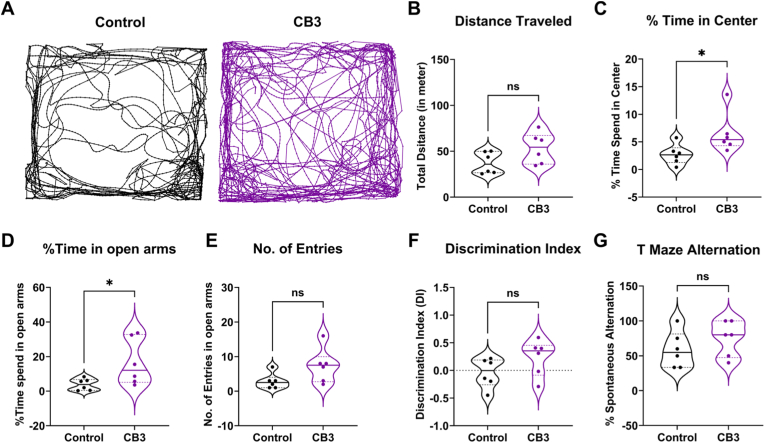
**Effect of CB3 treatment on epilepsy-associated cognitive deficits.** (A) Representative images of track path showing movement of control (black) and CB3 (purple) treated animals in open field arena two weeks after treatment. Open Field test: Total distance traveled (B), Percentage time spent in center (C) by vehicle and CB3 treated epileptic animals. Elevated plus maze test: Percentage time spent in open arms (D), No. of entries in open arms (E) by vehicle and CB3 treated epileptic animals. Novel object recognition test showing discriminative index (F) in vehicle and CB3 treated epileptic animals. T-maze spontaneous alteration test showing percentage of correct alteration (G) by vehicle and CB3 treated epileptic animals. n = 6 animals per group. Data are presented as mean ± s.e.m. Statistical significance was determined by unpaired *t*-test. ∗p < 0.05, ns: not significant.

Given that CB3 treatment can suppress spontaneous recurrent seizure activity and burden in the chronic phase, we next assessed whether these effects extended to behavioral outcomes. In contrast to early intervention after SE (cohort I), CB treatment in chronic phase did not improve locomotor activity, including total travel distance compared with the control group (Fig. 6B). However, CB3 treatment influenced anxiety-like behavior, as evidenced by the increased time spent in the center zone compared to control animals (Fig. 6C, p > 0.05) and showed more exploratory track patterns (Fig. 6A), changes that were not attributable to reduced locomotor activity. Similarly, in the elevated plus maze, CB3-treated animals spent significantly more time in the open arms (Fig. 6D, p < 0.05), although the number of open-arm entries did not differ between groups (Fig. 6E). To assess cognitive outcomes, we examined recognition memory and spatial learning. CB3 treatment did not alter discrimination index values in the novel object recognition test (Fig. 6F) or performance in the T-maze alternation task (Fig. 6G). These findings suggest that, while CB3 may attenuate anxiety-like behavior in the chronic phase of epilepsy, it does not reverse memory or learning deficits that are established during epileptogenesis.

## 4 Discussion

This study suggested that CB3, a thioredoxin-mimetic peptide, has properties consistent with a promising disease-modifying therapeutic approach in a preclinical model by disrupting processes that sustain epileptogenesis and chronic seizure activity. Epilepsy progression is strongly influenced by oxidative stress and the inflammatory cascade, which form a self-perpetuating cycle that exacerbates neuronal dysfunction and contributes to drug resistance [3,63]. By modulating these processes, CB3 treatment was associated with decreased seizure burden, preserved neuronal integrity, and partial improvement in selected behavioral outcomes across both early and chronic stages of experimental epilepsy.

In vitro, CB3 attenuated oxidative activity and proinflammatory cytokine expression associated with low-Mg^2+^ induced epileptiform activity (Fig. 1). While dihydroethidium (HEt) fluorescence is a widely used approach to assess redox imbalance; however, this method is not definitive because it cannot distinguish superoxide-specific products from non-specific oxidation events. As noted in the Consensus Statement on reactive species detection [64], fluorescence-based probes such as HEt produce overlapping signals from ethidium and 2-hydroxyethidium, which complicates interpretation, and HEt oxidation products can intercalate into DNA, further enhancing fluorescence and limiting specificity. For this reason, in the present study, HEt measurements are considered supportive indicators of oxidative dysregulation under epileptiform conditions rather than precise quantifications of superoxide generation. Nevertheless, the observed concentration-dependent reduction in HEt fluorescence, together with CB3's suppression of proinflammatory cytokines (IL-6, IL-1β, TNF-α) and upregulation of IL-10, is consistent with CB3's previously reported capacity to modulate oxidative and inflammatory pathways in various pathological conditions, such as cardiovascular diseases, allergic airway disease, and traumatic brain injury [32,33,38,39,52,53]. More specific approaches, such as LC-MS-based quantification of 2-hydroxyethidium or assays for lipid peroxidation products, will be needed in future studies to delineate the molecular mechanisms of CB3's redox activity.

In vivo, early intervention after SE (cohort I), CB3 significantly reduced seizure frequency, reduced seizure duration, delayed seizure onset, and lowered cumulative seizure burden (Fig. 2C–F), which was further supported by the greater cumulative probability of seizure-free days in CB3-treated animals than vehicle group (Fig. 2G and H). These findings suggest that CB3 may interfere with early pathological processes, distinguishing it from conventional ASMs that primarily suppress neuronal excitability without modifying disease progression [3]. Importantly, CB3 treatment was associated with preservation of locomotor activity and reduced anxiety-like behavior during epilepsy development (Fig. 3A–E). In addition, CB3-treated animals performed better in the T-maze spontaneous alternation task (Fig. 3G), indicating partial preservation of spatial working memory. However, we acknowledge the limitation that these behavioral assessments were not performed in naïve or sham-operated age-matched controls under identical experimental conditions. These behavioral improvements may reflect CB3-mediated preservation of hippocampal and prefrontal cortical function, regions critically involved in anxiety regulation and spatial working memory [65,66]. The hippocampus, particularly its CA1-CA3 subfields, plays a crucial role in spatial learning and working memory, while the medial prefrontal cortex (mPFC) modulates anxiety-like behaviors by interacting with the amygdala and hippocampus [[66], [67], [68], [69]]. These cognitive benefits may be related to reduced seizure-induced oxidative DNA damage in the hippocampus (Supp Fig. 2), although fluorescence-based markers such as 8-OHdG provide supportive rather than definitive measures [64]. In addition, protection of neuronal integrity within these regions (Fig. 4) further supports the possibility that CB3 may preserve networks essential for cognitive and emotional function, as oxidative stress and neuroinflammation are known to disrupt hippocampal plasticity and mPFC-amygdala communication during epileptogenesis [[70], [71], [72]]. Thus, CB3's redox and anti-inflammatory modulation may contribute to preserving functional circuitry supporting both cognitive and emotional domains. These behavioral benefits were accompanied by preservation of hippocampal neuronal integrity (Fig. 4), consistent with a neuroprotective association of CB3, and in line with previous reports that thioredoxin-based interventions support neuronal survival and cognitive outcomes in experimental models [32,37,39]. Recent work also highlights a role for PI3K/AKT signaling in anxiety regulation [73], providing possible mechanistic context for CB3's anxiolytic effects. However, as behavioral and histological measures were obtained at distinct experimental time points, performing correlation analyses between them would not be methodologically appropriate and was therefore not undertaken.

In the chronic phase of epilepsy (cohort II), CB3 treatment again led to a marked and sustained reduction in seizure frequency and overall seizure burden (Fig. 5). These therapeutic benefits persisted beyond the treatment period (Fig. 5B–E), which contrasts with conventional ASMs, which typically require continuous dosing and often lose efficacy because of pharmacoresistance [[74], [75], [76], [77]]. The ability of CB3 to modulate the redox signaling and inflammatory pathways may underlie its sustained effect, as previous studies have suggested that oxidative stress and neuroinflammation play a crucial role in seizure recurrence and drug-resistance [[5], [6], [7],^10^]. Recent findings further show that activation of the NLRP3 inflammasome can amplify both oxidative stress and neuroinflammatory signaling in chronic epilepsy, strengthening the link between these interacting pathological processes [28]. Interestingly, CB3 treatment in chronic epilepsy was associated with partial reduction in anxiety-like behavior (Fig. 6C and D) but did not interfere with recognition memory and spatial learning once deficits were established (Fig. 6F and G). This observation is consistent with prior studies indicating that prolonged seizures cause irreversible hippocampal injury, limiting the potential for cognitive recovery in chronic epilepsy [78,79]. In addition, we assessed canonical oxidative stress–related markers, including glutathione redox status (GSH, GSSG, and their ratios) and superoxide dismutase (SOD) activity (Supp Figs. 3 and 4, respectively). No significant differences were observed in either glutathione redox parameters or SOD activity in the hippocampus or cortex of CB3-treated animals compared with controls, which may reflect the delayed experimental endpoint and suggests that CB3-induced redox modulation occurs primarily during the treatment window rather than as a sustained change in basal antioxidant capacity. Together, these findings indicate that CB3 may be more effective in preventing seizure recurrence and protecting neuronal integrity, with cognitive benefits being more evident when treatment is introduced at earlier stages.

Given its mechanism of reducing oxidative stress and neuroinflammation, CB3 may have the potential to complement existing ASMs. Most ASMs suppress neuronal excitability but do not address these underlying pathological drivers, which can lower seizure threshold and contribute to pharmacoresistance [4]. Thus, combining CB3 with standard ASMs could potentially enhance seizure control, reduce required drug doses, or improve tolerability. Although this study did not evaluate combination therapy, exploring CB3 as an adjunct treatment represents an important direction for future work, particularly in drug-resistant epilepsy.

Compared with other antioxidant-based therapies, CB3 appears to confer unique advantages. N-acetylcysteine (NAC) and its amide derivative, N-acetylcysteine amide (AD4/NACA) have been tested for its therapeutic efficacy in epilepsy. Although NAC is known to provide neuroprotective benefits, its poor blood‒brain barrier (BBB) permeability and low bioavailability restrict its therapeutic efficacy in chronic neurological conditions, such as epilepsy [37,80,81]. This is supported by recent findings showing that NAC effectively reduces ROS-induced oxidative stress, apoptosis, and cell-cycle arrest in vitro, demonstrating its antioxidant capacity [82] but also underscoring the need for agents such as CB3 that additionally modulate neuroinflammatory pathways. In contrast, AD4/NACA was developed to overcome these limitations to improve BBB penetration [83], has demonstrated enhanced antioxidant efficacy than NAC, yet its impact on seizure modulation remains inconclusive and safety data in humans are limited. In contrast, CB3-contains two cysteine residues instead of one as in AD4/NACA, can generate a cluster of cysteine-rich proteolytic fragments that exert broader and more potent redox effects. Moreover, CB3 modulates both the thioredoxin redox system and neuroinflammatory pathways [37], and in this study was associated with seizure suppression that persisted after treatment withdrawal. This sustained effect contrasts with many conventional ASMs that require continuous dosing and may lose efficacy with prolonged use [[74], [75], [76], [77]]. Together, these findings support the view that CB3 may represent a dual redox and anti-inflammatory modulator with potential disease-modifying properties, though comprehensive pharmacokinetic and safety evaluations are warranted to support clinical translation. Furthermore, CB3 primarily acts through modulation of the TrxR/Trx system, a central regulator of cellular redox balance, antioxidant defense, apoptosis, and inflammatory signaling [84,85]. Beyond its redox-related activity, CB3 also attenuates neuroinflammatory responses, which distinguishes it from other redox-based interventions. Anti-inflammatory agents such as minocycline and nonsteroidal anti-inflammatory drugs (NSAIDs), including cyclooxygenase-2 (COX-2) selective inhibitors (COXIBs), have been investigated in epilepsy; however, their efficacy has been inconsistent, and long-term safety concerns have limited their clinical utility, with several COXIBs (rofecoxib, valdecoxib, lumiracoxib) withdrawn from the market [86]. Many of the remaining agents continue to carry regulatory warnings from the FDA, although some are still licensed in the European Union [87,88]. In this context, the dual ability of CB3 to modulate both oxidative and inflammatory pathways supports its candidacy as a disease-modifying approach for epilepsy.

A limitation of this study is that oxidative activity was assessed using fluorescence-based detection, which, although widely used, lacks specificity for individual reactive species and should therefore be interpreted as supportive rather than definitive evidence of redox dysregulation [64]. Additional approaches such as LC-MS-based quantification will be important for mechanistic clarification in future studies. Future studies will be required to define the optimal dosing strategy and therapeutic window for CB3, including dose-response relationships and treatment duration, to guide its clinical translation. In addition, the acute anticonvulsant effects of CB3 were not evaluated, as the in vivo study focused on sustained treatment outcomes, and should be addressed in future studies specifically designed to assess immediate seizure suppression. Further work is also needed to establish the pharmacokinetics and safety profile of CB3 in vivo. In addition, because the present study included only male rats, sex-specific differences in CB3 efficacy remain unknown and should be evaluated in future studies using both sexes to determine whether CB3 exerts comparable antiepileptogenic, antiseizure, and behavioral effects.

## 5 Conclusion

Our findings suggest that **TXM-CB3** is associated with reduced seizure burden, preservation of neuronal integrity, and partial attenuation of epilepsy-associated behavioral impairments in both epileptogenesis and chronic epilepsy models. By targeting the pathological processes that drive seizure recurrence and disease progression, **TXM-CB3** represents a therapeutic strategy mechanistically distinct from conventional antiseizure medications. Given its **dual activity** and **sustained effect in preclinical model**s, further studies should focus on **additional preclinical validation and careful clinical translation**, with an emphasis on optimizing **dosing regimens** and evaluating **long-term safety profiles** to assess the potential of **TXM-CB3** as a **candidate with disease-modifying potential in epilepsy**.

## Ethics approval

All animal experiments were conducted in compliance with the guidelines of the Association for Assessment and Accreditation of Laboratory Animal Care International and were approved by the Institutional Animal Care and Use Committee (IACUC) of Hebrew University of Jerusalem (Protocol Code: MD-20-16254-5).

## Funding

This study was supported by funding from 10.13039/501100003977The Israel Science Foundation (grant no. 1976/20, awarded to TSA) and the 10.13039/501100006245Ministry of Science and Technology, Israel (grant no. 5100, awarded to TSA).

## CRediT authorship contribution statement

**Prince Kumar Singh:** Formal analysis, Investigation, Methodology, Project administration, Visualization, Writing – original draft, Writing – review & editing. **Shweta Maurya:** Formal analysis, Investigation, Methodology, Writing – review & editing. **Aseel Saadi:** Methodology, Writing – review & editing. **Sereen Sandouka:** Methodology, Writing – review & editing. **Taige Zhang:** Methodology. **Orya Kadosh:** Methodology, Writing – review & editing. **Yara Sheeni:** Writing – review & editing. **Valeria Martin:** Methodology, Writing – review & editing. **Daphne Atlas:** Conceptualization, Writing – review & editing. **Tawfeeq Shekh-Ahmad:** Conceptualization, Funding acquisition, Project administration, Supervision, Visualization, Writing – original draft, Writing – review & editing.

## Declaration of competing interest

The authors declare that they have no known competing financial interests or personal relationships that could have appeared to influence the work reported in this paper.

## Data Availability

Data will be made available on request.
