# Supplementary material for: Thioredoxin-mimetic peptide attenuates epilepsy progression and neurocognitive deficits
Source: Redox Biol. 2026 Jan 10;90:104021. doi: 10.1016/j.redox.2026.104021 (PMC12830260; doi:10.1016/j.redox.2026.104021)
Supplement: Multimedia component 1 [file mmc1.docx]

**Thioredoxin-Mimetic peptide attenuates epilepsy progression and neurocognitive deficits**

Prince Kumar Singh^#1^, Shweta Maurya^#1^, Aseel Saadi^1^, Sereen Sandouka^1^, Taige Zhang^1^, Orya Kadosh^1^, Yara Sheeni^1^, Valeria Martin^2^, Daphne Atlas^2^, Tawfeeq Shekh-Ahmad^1^*

^1^The Institute for Drug Research, The School of Pharmacy, Faculty of Medicine, The Hebrew University of Jerusalem, Jerusalem, Israel – 91120.

^2^The Alexander Silberman Institute of Life Science, The Hebrew University of Jerusalem, Jerusalem, Israel.

^#^These authors contributed equally to this manuscript

*Corresponding author: Tawfeeq Shekh-Ahmad, The Institute for Drug Research, The School of Pharmacy, Faculty of Medicine, The Hebrew University of Jerusalem, Jerusalem, Israel – 91120.

E-mail: [Tawfeeq.Shekh-Ahmad@mail.huji.ac.il](mailto:Tawfeeq.Shekh-Ahmad@mail.huji.ac.il)

**
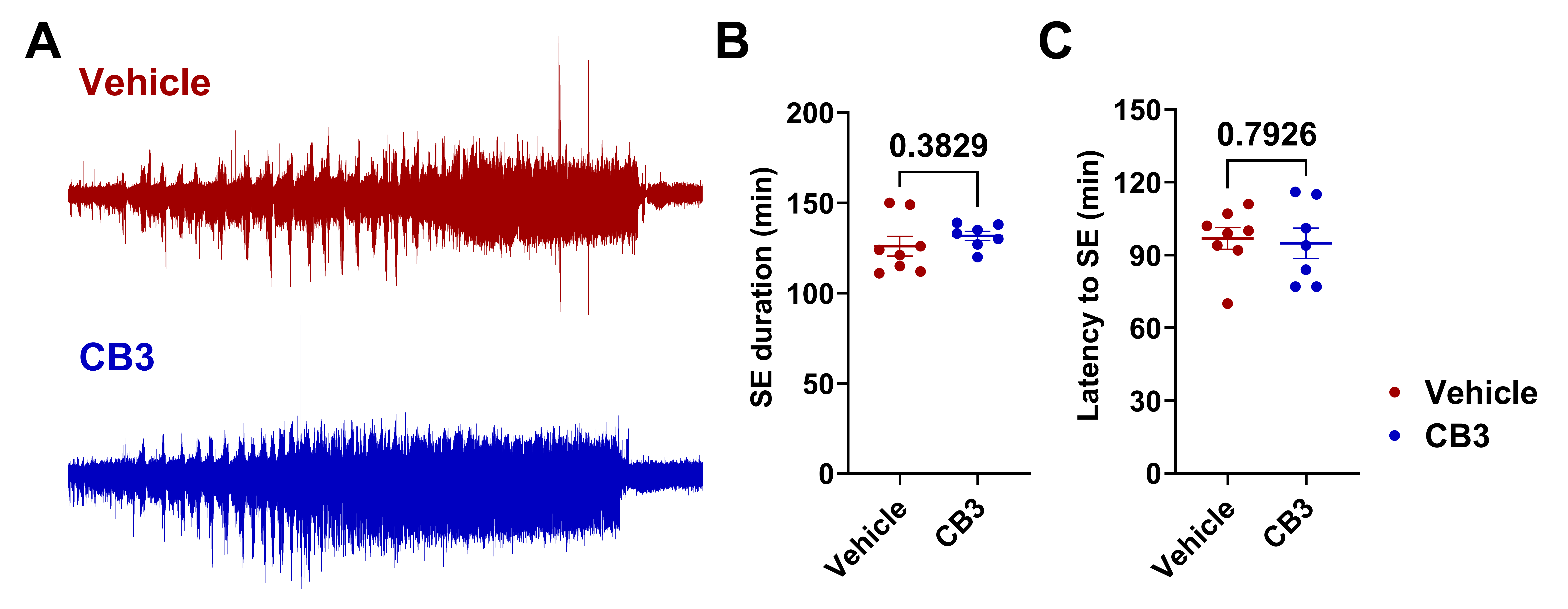
**

**Supp Figure 1. Induction of status epilepticus in Cohort I animals.**

(A) Representative EEG traces showing the induction of SE following KA administration in animals allocated to the Vehicle (red) and CB3 (blue) groups in Cohort I. (B) Comparison of SE duration between animals in the Vehicle and CB3-treated groups. (C) Latency to SE onset following KA injection in the two allocated groups. n = 8 animals (Vehicle) and 7 animals (CB3). Data are presented as mean ± s.e.m. Statistical significance was assessed using an unpaired t-test. ns, not significant (p > 0.05).

**Supp Figure 2. Effect of CB3 treatment on seizure induced oxidative DNA damage in hippocampal neurons.**

Representative immunofluorescence images of CA1 (A), CA3 (B) hippocampal subfields of vehicle or CB3 treated animals after SE, followed by 12 weeks of vECoG monitoring. Sections were stained for NeuN (green, neuronal marker), DAPI (blue, nuclear marker), and 8-OHdG (red, marker of oxidative DNA damage). Merged images show co-localization of 8-OHdG with NeuN, indicating neuronal oxidative stress. (C-D) Quantification of 8-OHdG means fluorescence intensity (arb. unit) in either CA1 (C) and CA3 (D) subfields of the hippocampus. n=5 animals per group. Scale bars: 100 μm. Data are presented as mean ± s.e.m. Statistical significance was determined by unpaired t test. *p<0.05, ns: not significant.


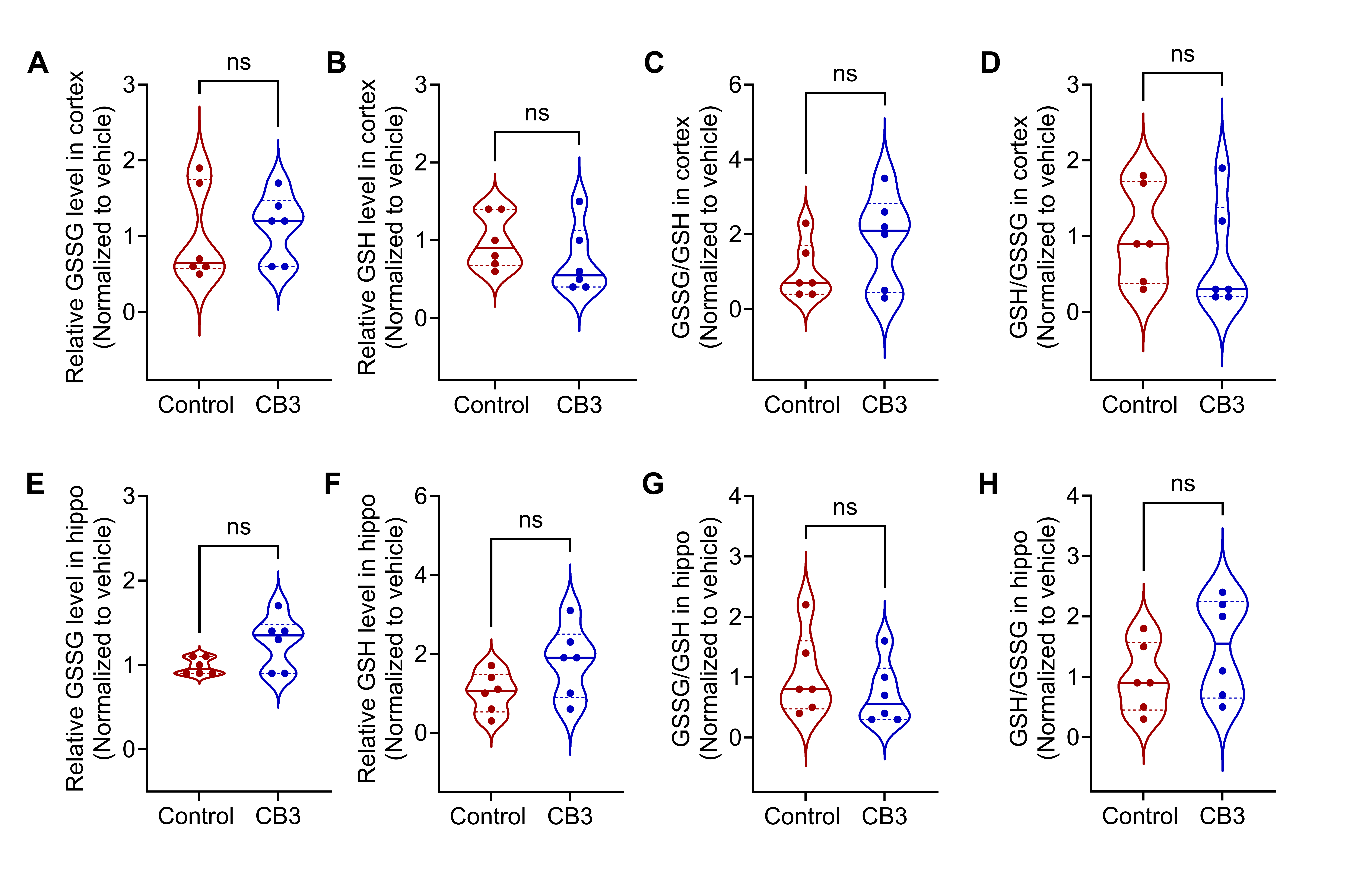


**Supp Figure 3. Effects of CB3 treatment on glutathione redox status in chronic epilepsy.**

Relative levels of oxidized glutathione (GSSG; A, E), reduced glutathione (GSH; B, F), GSSG/GSH ratio (C, G), and GSH/GSSG ratio (D, H) were measured in the cortex (A-D) and hippocampus (E-H) of animals with chronic epilepsy treated with vehicle (control) or CB3. Brain tissues were collected 4 weeks after treatment withdrawal, following continuous ECoG monitoring, representing the experimental endpoint (Figure 5A). Glutathione levels were quantified using an enzymatic recycling assay and normalized to the mean value of the vehicle-treated controls (control = 1). Data are presented as mean ± s.e.m. n=6 animals per group. Statistical significance was determined using an unpaired t-test. ns, not significant.

**Assessment of glutathione redox status**

Total glutathione (GSH + GSSG) was quantified using a glutathione reductase-based enzymatic recycling assay (Cayman Chemical Glutathione Assay Kit, Item No. 703002) according to the manufacturer’s instructions. Samples were deproteinated with metaphosphoric acid, neutralized with triethanolamine, and assayed colorimetrically using DTNB, with absorbance measured at 405-414 nm. Reduced (GSH) and oxidized (GSSG) glutathione levels and their ratios were calculated from a standard curve and normalized to the vehicle-treated group.


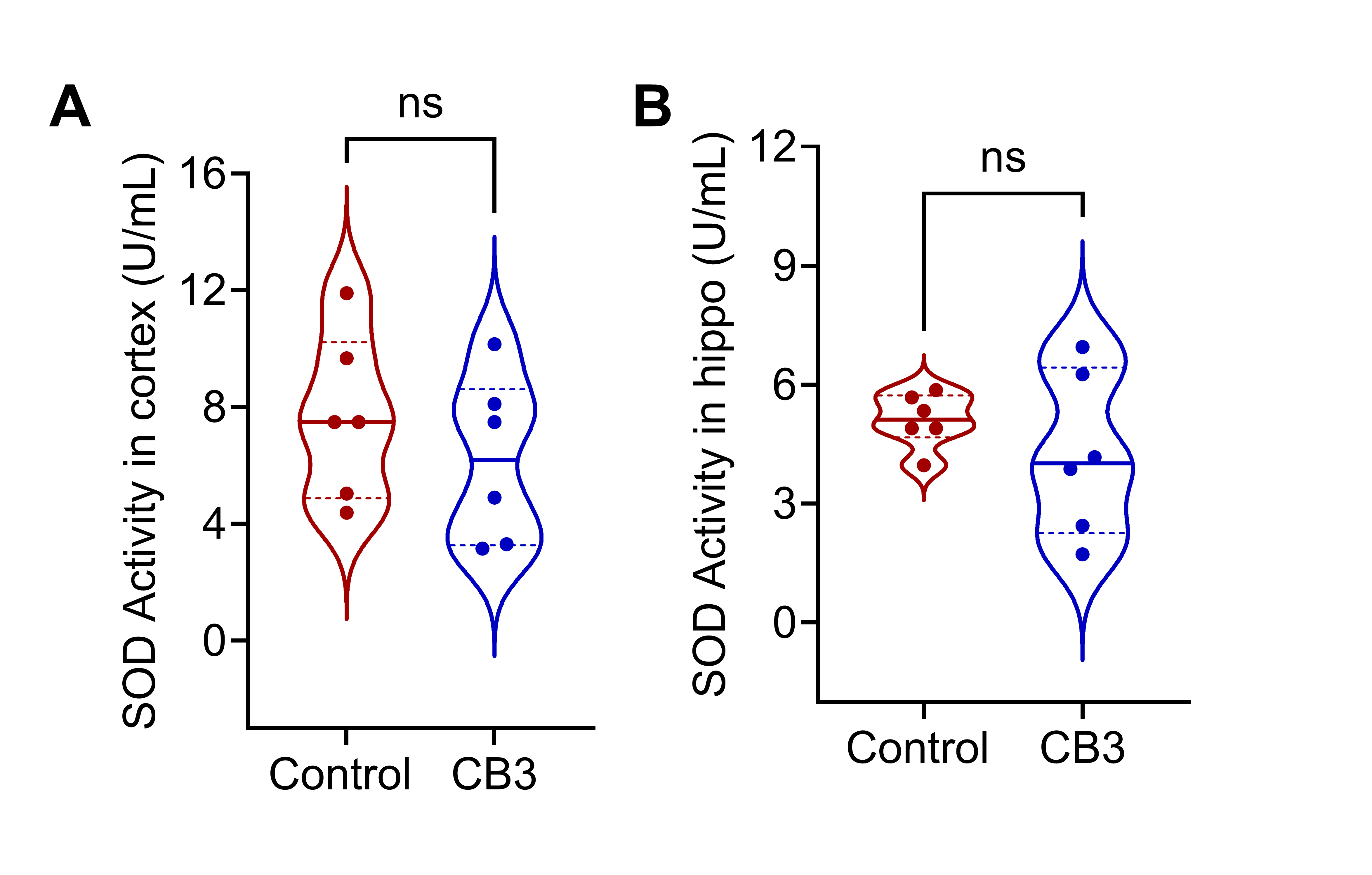


**Supp Figure 4. Effects of CB3 treatment on superoxide dismutase activity in chronic epilepsy.**

Superoxide dismutase (SOD) activity was measured in the cortex (A) and hippocampus (B) of animals with chronic epilepsy treated with vehicle (control) or CB3. Brain tissues were collected 4 weeks after treatment withdrawal, following continuous ECoG monitoring, representing the experimental endpoint (Figure 5A). SOD activity was quantified using a colorimetric assay based on inhibition of tetrazolium salt reduction by superoxide radicals and is expressed as units per milliliter (U/mL). Data are presented as mean ± s.e.m. n=6 animals per group. Statistical significance was determined using an unpaired t-test. ns, not significant.

**Assessment of superoxide dismutase activity**

Superoxide dismutase (SOD) activity was measured using a colorimetric assay based on inhibition of tetrazolium salt reduction by superoxide radicals generated by xanthine oxidase (Cayman Chemical, Item No. 706002), following the manufacturer’s protocol. Absorbance was measured at 450 nm, and SOD activity was calculated from a standard curve and expressed as units per milliliter, where one unit corresponds to 50% dismutation of superoxide radicals.
